# The Impact of Genetic Variations in ADORA2A in the Association between Caffeine Consumption and Sleep

**DOI:** 10.3390/genes10121021

**Published:** 2019-12-06

**Authors:** Mégane Erblang, Catherine Drogou, Danielle Gomez-Merino, Arnaud Metlaine, Anne Boland, Jean François Deleuze, Claire Thomas, Fabien Sauvet, Mounir Chennaoui

**Affiliations:** 1 Unité Fatigue et Vigilance, Institut de Recherche Biomédicale des Armées (IRBA), EA 7330 VIFASOM, Université de Paris, 75004 Paris, France; megane.erblang@gmail.com (M.E.); catherine.drogou@gmail.com (C.D.); dangomez51@gmail.com (D.G.-M.); fabien.sauvet@gmail.com (F.S.); 2 EA 7330 VIFASOM, Université de Paris, APHP, Hôtel Dieu, Centre du Sommeil et de la Vigilance, 75004 Paris, France; arnaud.metlaine@aphp.fr; 3 Centre National de Recherche en Génomique Humaine (CNRGH), Institut de Biologie François Jacob, CEA, Université Paris-Saclay, 91057 Evry, France; boland@cng.fr (A.B.);; 4 Unité de Biologie Intégrative des Adaptations à l’Exercice, Université Evry, Université, Paris-Saclay, 91025 Evry, France; Claire.thomas@uni-evry.fr

**Keywords:** caffeine, sleep, adenosine A2A receptor, ADORA2A, polymorphisms

## Abstract

ADORA2A has been shown to be responsible for the wakefulness-promoting effect of caffeine and the 1976T>C genotype (SNP rs5751876, formerly 1083T>C) to contribute to individual sensitivity to caffeine effects on sleep. We investigate the association between six single nucleotide polymorphisms (SNP) from ADORA2A and self-reported sleep characteristics and caffeine consumption in 1023 active workers of European ancestry aged 18–60 years. Three groups of caffeine consumers were delineated: low (0–50 mg/day, less than one expresso per day), moderate (51–300 mg/day), and high (>300 mg/day). We found that at caffeine levels higher than 300 mg/day, total sleep time (TST) decreased (*F* = 13.9, *p* < 0.01), with an increase of insomnia (ORa [95%CI] = 1.5 [1.1–1.9]) and sleep complaints (ORa [95%CI] = 1.9 [1.1–3.3]), whatever the ADORA2A polymorphism. Odds ratios were adjusted (ORa) for sex, age, and tobacco. However, in low caffeine consumers, lower TST was observed in the T allele compared to homozygote rs5751876 and rs3761422 C carriers. Conversely, higher TST was observed in rs2298383 T allele compared to C and in rs4822492G allele compared to the homozygote C (*p* < 0.05). These 4 SNPs are in strong linkage disequilibrium. Haplotype analysis confirmed the influence of multiple ADORA2a SNPs on TST. In addition, the rs2298383 T and rs4822492 G alleles were associated with higher risk of sleep complaints (Ora = 1.9 [1.2–3.1] and Ora = 1.5 [1.1–2.1]) and insomnia (Ora = 1.5 [1.3–2.5] and Ora = 1.9 [1.3–3.2). The rs5751876 T allele was associated with a decreased risk of sleep complaints (Ora = 0.7 [0.3–0.9]) and insomnia (Ora = 0.5 [0.3–0.9]). Our results identified ADORA2A polymorphism influences in the less-than-300-mg-per-day caffeine consumers. This opens perspectives on the diagnosis and pharmacology of sleep complaints and caffeine chronic consumption.

## 1. Introduction

Consumption of caffeine, an adenosine receptor antagonist, is the most widely consumed stimulant in the world [1,2]. It is found in a variety of foods and beverages, such as coffee, tea, chocolate, and energy drinks; coffee being the primary dietary caffeine source in Western Europe and the United States. It is used to enhance performance by athletes and as a countermeasure against fatigue in shift workers, airline pilots, and truck drivers [3]. Caffeine attenuates waking and sleep electroencephalographic markers of sleep homeostasis in humans [4].

It is generally accepted that caffeine promotes wakefulness by unselectively antagonizing adenosine receptors in the brain [2,5]. Adenosine is a metabolic intermediate of the energy-rich molecule adenosine-tri-phosphate (ATP), acting as an endogenous homeostatic sleep factor, with increased levels in the basal forebrain during prolonged wakefulness and resolves during sleep [6]. The sleep-inducing effects of extracellular adenosine are mediated mainly through the inhibitory G protein-coupled adenosine A1 receptor and the excitatory G protein-coupled adenosine A2A receptor (A2AR) that are ubiquitously distributed throughout the brain [5]. Accumulating evidence suggests that among these receptors responsible for sleep induction, the role of A2A is predominant in sleep regulation, whereas A1 contributes to sleep induction in a region-dependent manner but may not be absolutely necessary for sleep homeostasis [7]. In our previous study, we described an upregulation of A2AR expression in human leukocytes from healthy young subjects after 24 h of sleep deprivation while A1R was not changed [8]. The metabolism, clearance, and pharmacokinetics of caffeine is affected by many factors (age, smoking, diet, medications, sex and hormones, liver disease, obesity) and also by genetic variability affecting caffeine levels and effects at the pharmacokinetic and pharmacodynamic levels that both critically drive the level of caffeine consumption [9]. Both at pharmacodynamic and consumption levels, and those of tolerance to sleep deprivation and side effects, several polymorphisms of the ADORA2A gene, the major target of caffeine action in the brain, have been shown to be implicated [10,11,12,13,14].

The ADORA2A locus is located on human chromosome 22 and contains a set of SNPs (single nucleotide polymorphism), the most studied being rs5751876 (formally designated as 1976C/T or 1083C/T). Other polymorphisms such as rs2298383 and rs3761422 are in high linkage disequilibrium with the rs5751876 [12]. The 1976T>C polymorphism of ADORA2A modulates the EEG activity of the wake–sleep continuum [15], contributes to individual sensitivity to caffeine effects on sleep [11] and greater sensitivity to caffeine-induced anxiety [12]. Recently, an association was found between caffeine consumption and objective sleep variables in the T allele genotype carriers and not in the CC genotype of the ADORA2A rs5751876 single nucleotide polymorphism (SNP) [16]. Furthermore, among several ADORA2A polymorphisms, rs5751876 and several other variants in high LD (linkage disequilibrium) (rs5751862, rs2298383, and rs3761422), as well as the corresponding haplotypes, were found associated with anxiety, and of these variants, rs2298383 was shown to possess functional potential and might, therefore, represent the true underlying causal variant [17]. In a second study, these authors showed that the rs5751876 SNP is highly correlated with in vivo adenosine A1 receptor binding in the human brain [18].

Our aim is to assess the influence of six ADORA2A SNPs on self-reported sleep characteristics and caffeine consumption in a French active worker population (*n* = 1023 participants). The six ADORA2A SNPs, selected because of their involvement in caffeine consumption, sensitivity to caffeine effects on sleep, sleep disorders and anxiety in literature, were rs5751876, rs2298383, rs3761422, rs5751862, rs2236624, and rs4822492.

The principal conclusions are that: (1) in low caffeine consumers (less than 300 mg per day) a combination of ADORA2A polymorphisms influences TST (total sleep time) and the risk of sleep complaints and insomnia, and (2) at caffeine daily consumption higher than 300 mg/day, total sleep time (TST) decreases and prevalence of insomnia and sleep complaints increases, whatever the ADORA2A polymorphism. This opens perspectives on the diagnosis and pharmacology of sleep complaints and caffeine chronic consumption. 

## 2. Results

### 2.1. Subjects

The questionnaire was completed by 1083 participants of European ancestry. We excluded 60 participants, of which 22 provided a saliva sample that was not usable, one did not sign the informed consent, 34 had at least one missing response on the questionnaire, and 3 presented an exclusion criteria. Finally, a total of 1023 questionnaires (618 men and 405 women) were analyzed.

### 2.2. Sociodemographic Data and Lifestyle Habits

The participants were aged between 18 and 60 years (32.5 ± 9.6) and were divided into 60.4% (*N* = 618) male and 39.6% (*N* = 405) female (Table 1). Most of the participants (43.3%) were single, and 56.6% were married or living with a partner. About 37.8% of them had children. The mean BMI was 23.6 ± 3.5 kg/m^2^; 56 (5.5%) were obese and 225 (22.0%) were overweight. About 192 (18.8%) of the participants are current smokers and 81.0% have never been smokers. About 76 (7.4%) of the participants consumed alcohol and 671 (65.6%) exercised more than 2 h per week. 129 (12.6%) regularly took pharmaceuticals treatments. The most frequent medications were contraceptive (26.5%), levothyrox (7.5%), blood pressure (7.5%), allergy (7.4%), asthma (7.5%) and proton pump inhibitor (PPI, 6.5%) treatments. The use of sedatives concerned only 9 participants (i.e., 0.9%).

The mean daily caffeine consumption was 243 ± 208 (SD) mg/d. Two hundred and two (19.7%) pertain to the low (0 to 50 mg/day) caffeine consumer group, 478 (46.7%) to the moderate (51 to 300 mg/day), and 343 (33.5%) to the high (>300 mg/day) caffeine consumers. Age varied among the groups, with younger subjects in low compared to high caffeine consumers (28.7 ± 8.7 years vs. 35.9 ± 9.1 years). Smokers were overrepresented (*p* < 0.01) in the high (30.8%) and moderate (12.1%) caffeine consumption groups compared to the low caffeine group (7%). In the moderate and the high caffeine consumer groups, 5.5% and 19.0% of smokers consumed more than 5 cigarettes per day, respectively. Of the 1023 participants, 46.8% reported sleep complaints and 10.7% insomnia.

### 2.3. Sleep Duration, Sleep Complaints and Insomnia According to Caffeine Consumption

The self-reported nocturnal total sleep time (TST) significantly decreased with the increase of caffeine consumption, whatever the genotype. The ANOVA analysis showed that TST was significantly lower in high consumers compared with low and moderate consumers (*F* = 13.85, *p* < 0.01; Table 2).

Sleep complaints were significantly higher (ORa = 1.9 [1.1–3.3]) in high caffeine consumers compared to low and moderate consumers, and insomnia was higher in high compared to low consumers (ORa = 1.5 [1.1–1.9]).

The percentage of participants reporting caffeine side effects significantly increased with the daily caffeine consumption (*p* < 0.01), with 50% reporting at least one side effect when caffeine consumption is higher than 300 mg. The diuretic effect of caffeine is prevalent when caffeine consumption is higher than 300 mg.

### 2.4. Genotype Prevalence of the Six ADORA2A Polymorphisms

**Figure 1:**
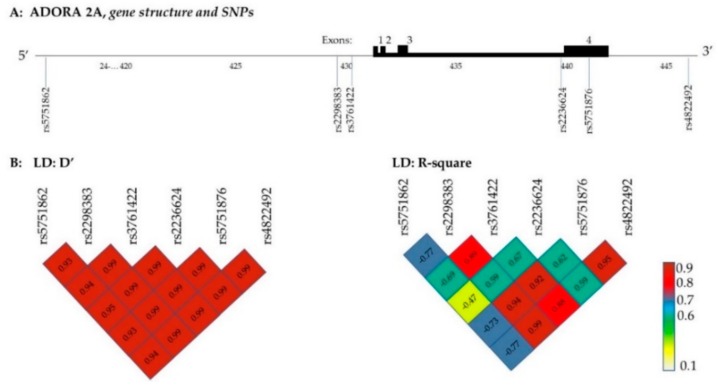
Genomic organization of the ADORA2A gene (NM_000675 located on Chr. 22q11.23, GRCh38.p12) with the position of selected SNPs (**A**) and pairwise linkage disequilibrium (LD) structure (**B**). ADORA2A coding exons are 1, 2, 3, 4, and illustrated by higher black block. On the GRCh38 reference genome, the rs5751862 is on 5’regulation, rs2298383 and rs3761422 are 5’UTR, rs2236624 is intronic on 3-4, rs5751876 is a synonymous variant located on the exon 4, and rs4822492 is on 3’UTR AS. Shades of colored/numbers in boxes show the extent of LD (red/higher numbers = higher LD, white/lower numbers = lower LD) assessed through statistics D’ and *R*-square.

The genotype prevalence of the six ADORA2A SNPs in our study is similar to the 1000 Genomes Project data on the GRCh38 reference assembly (Table 3). Genotype frequencies of all SNPs conformed to the Hardy–Weinberg equilibrium (*p* > 0.16 for all). The pairwise linkage disequilibrium analysis showed moderate to strong LD between ADORA2A variants (Figure 1B). We observed a *R*-square value >0.8 between rs2298383, rs3761422, rs5751876, and rs4822492.

### 2.5. Impact of the Six ADORA2A SNPs on Total Sleep Time (TST) According to Caffeine Consumption

The two-way ANOVA analysis showed no significant effect of genotype but a significant effect of caffeine consumption on TST for the six ADORA2A SNPs, with significant interaction between genotype and caffeine consumption for 4 SNPs (Table 4 and Table 5). Post hoc analysis showed that genotype is associated with a significant difference in TST only for low caffeine consumers (<50 mg per day, i.e., less than one expresso coffee per day). In low caffeine consumers, TST level was significantly lower in the T/C and T/T carriers compared to C/C (ancestral) carriers of two ADORA2A SNPs (rs5751876 and rs3761422) while TST level was higher in the T/T (vs. C/C) carriers of the rs2298383 and the G (G/C and G/G) compared to C/C carriers of the rs4822492 (Table 5).

### 2.6. Impact of ADORA2A Haplotypes on Total Sleep Time (TST) According to Caffeine Consumption

Six (1 to 6) different haplotypes (ATCCCG, GCTTTC, GCTCTC, GTCCCG, GCCCTC, GCCCCC) and one Rare were found, the higher frequency being 45.3% for haplotype 1 (Table 6). The haplotypes 2 (21.2%) and 3 (15.7%) in our study respectively corresponded to the haplotypes HT1 and HT4 in the Bodenmann et al. (2012) study [14]. In the Bodenmann study, HT4 haplotype carriers consistently performed at a higher level on a PVT task during sleep deprivation and presented less rebound of SWA (slow-wave activity) in recovery sleep than non-HT4 carriers.

**Figure 2:**
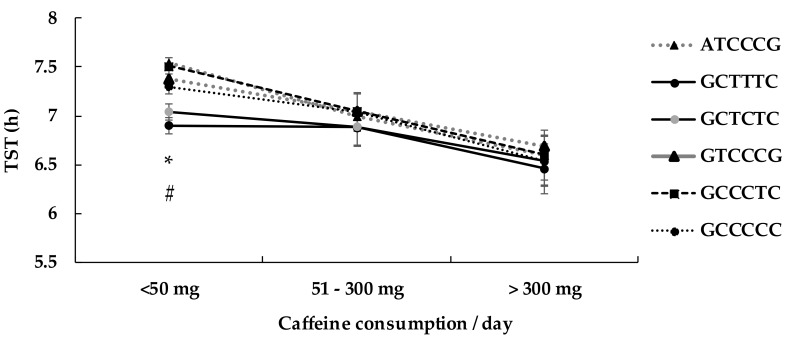
Impact of the six haplotypes of ADORA2A on total sleep time (TST).Values are mean ± 95% CI; * difference (*p* < 0.005) between GCTTTC and ATCCCG haplotype 1 (45.3%); # between GTCCCCG and ATCCCG (*p* < 0.05).

The two-way (caffeine and haplotype) ANOVA analysis showed no significant effect of haplotype (*p* = 0.10; *F* = 1.42) but a significant effect of caffeine consumption (*p* = 0.02; *F* = 4.51) on daily TST, with a significant interaction (*p* = 0.03; *F* = 2.42). All haplotypes are associated with a significant effect of caffeine consumption, with the lower TST level in high consumers in comparison with low consumers (Table 7). In addition, the TST was significantly lower in low caffeine consumers in the haplotypes 2 and 3 (GCTTTC and GCTCTC) compared with the most frequent haplotype 1 (ATCCCG) (Table 7, Figure 2). In moderate compared with low caffeine consumers, TST was not significantly different in the haplotypes 2 and 3 (GCTTTC and GCTCTC), whereas it was significantly lower for four other haplotypes (1 and 4 to 6).

### 2.7. Impact of the Six ADORA2A SNPs on Sleep Complaints and Insomnia According to Caffeine Consumption

The risk of sleep complaints was significantly lower in the C/T and T/T compared to C/C (ancestral) genotype carriers for rs5751876 in moderate caffeine consumers and all subjects. However, in moderate and in all subjects, the risk of sleep complaints was higher in T/T compared to C/C for rs2298383 and in G/G compared to C/C (ancestral) genotype carriers for rs4822492 in moderate and in all subjects (Table 4 and Table 8).

The results on the impact of six ADORA2A SNPs on reported insomnia, according to the three groups of caffeine consumers, are not shown because less than 10% of subjects were concerned and the numbers of subjects carrying each of the genotype SNPs in the different caffeine groups were low. However, if we consider all subjects, the risk of insomnia is significantly higher for the T/T and G/G compared to C/C ancestral genotype carriers of rs2298383 and rs4822492, respectively (Table 4).

## 3. Discussion

In the present study, we investigate the effect of six genetic polymorphisms of ADORA2A on sleep (duration and disorders) and the association with caffeine consumption groups (low, moderate, and high consumers) in 1023 French active workers aged between 18 and 60 years. We selected polymorphisms that were associated previously with the interindividual variability in brain electrical activity during sleep and wakefulness [14,15], with the individual sensitivity to subjective and objective caffeine effects on sleep [11,19], anxiogenic response to caffeine [12,20], with caffeine consumption [10], with individual differences in anxiety-related personality and panic disorder [17], and sleep disturbances [19,21]. We delineate three groups of caffeine consumers according to several criteria. There is a consensus that the daily ingestion of 300–400 mg caffeine (around 4–5 cups of expresso coffee) does not raise any health concern, and that high doses of caffeine (400–800 mg in one sitting) may have negative effects (anxiety, nervousness, insomnia, tachycardia, and trembling) [22]. Moreover, 300 mg is also the dose of caffeine contained in the pharmacological form of the slow-release caffeine that was shown to be an advisable alternative to other psychostimulants for long work schedules, and night shifts [23]. The low caffeine consumers (0–50 mg/day) were in accordance with Rogers et al. (2010) [12].

Our study focuses on healthy and active workers of European ancestry, excluding retired and medical workers, who are neither overweight nor obese according to WHO criteria, who practice physical activity for the majority, consume little alcohol, with very few smokers. As the genotype frequency analysis shows, our population is very similar to the 1000 Genomes project population [24].

The significant impact of genetic variants on TST is only shown in low caffeine consumers (0–50 mg, corresponding to less than one expresso coffee per day). Several genetic mutations are associated with lower or higher TST. Individuals carrying T allele (homozygous or heterozygous) of rs5751876 and rs3761422 exhibit a significant lower TST whereas T allele of rs2298383 is associated with higher TST when compared to homozygous C ancestral allele carriers. In addition, TST is higher for the G allele (homozygous or heterozygous) carriers of rs4822492 compared to the ancestral homozygote C/C. The haplotype analysis revealed a significant lower TST for two haplotypes (2 and 3) combining T allele carriers of rs5751876 and rs3761422, and C allele carriers of rs2298383 and rs4822492, in comparison to the more frequent haplotype 1 (45.3%). Furthermore, TST was significantly lower in high caffeine consumers (>300 mg per day) compared to low and moderate, regardless of the selected ADORA2A polymorphism.

Secondly, our results showed that the risk of sleep complaints and insomnia is higher in participants carrying mutations on rs2298383 and rs4822492 (after adjustment for age, tobacco, and sex) in moderate and all caffeine consumers, but not in high consumers. There were significantly more subjects with sleep complaints in the high caffeine group (52.5%) than in the two other groups, and more subjects with insomnia comparatively with the low caffeine group (12.5 % vs. 7.4%).

Therefore, biological follow-up of genetic variants of the G-protein coupled A_2A_ receptor can provide additional insights into molecular processes underlying sleep duration and disorders, valuable for enhancing scientific knowledge of sleep and for informing new therapeutic approaches. Subsequent pharmacological studies from several laboratories have demonstrated that adenosine and its receptor agonists promote, but antagonists such as caffeine inhibit, both NREM and REM sleep stages [25]. There are four G protein-coupled adenosine receptor subtypes, A_1_, A_2A_, A_2B_, and A_3_ [26], and accumulated findings indicate a predominant role of A2AR in sleep regulation and caffeine-induced wakefulness [7,27]. We previously showed that one night of sleep deprivation induced an up-regulation of the A2AR gene expression in leukocytes from healthy subjects [28].

The genetic variation of ADORA2A was found to be an important determinant of wake promotion by caffeine, particularly the rs5751876 polymorphism [11,29]. Using polysomnography EEG in healthy males in the recovery night after sleep deprivation, Retey et al. [11] observed that caffeine increases spectral power in the beta band (16.625–20.125 Hz) in C/C compared to T/T genotype carriers of the ADORA2A c.1083T/C genotype (also designated 1976C/T or rs5751876 now). As in our study, the C/C genotype for rs5751876 seems associated with an increased likelihood of being sensitive to caffeine and an increased likelihood of insomnia when exposed to caffeine. Moreover, caffeine was shown to attenuate the rebound of SWA (slow-wave activity) occurring in the recovery night following sleep deprivation in subjects with non-HT4 haplotype ADORA2A only (this does not occur in HT4) [14]. Our results, in particular, the haplotype analysis, confirm that caffeine sensitivity is associated with several SNP mutations. We showed that the haplotypes carriers which are insensitive to caffeine effects on sleep were T allele carriers for rs5751876 and rs3761422, while being C carriers for rs4822492 and rs2298383. This profile is similar to the HT4 profile described by Bodenmann et al. [14] and confirms that T allele carriers of rs5751876 seem insensitive to caffeine effects on sleep duration [11,14]. However, Nunes et al. 2017, with a polysomnographic study, failed to demonstrate an association between ADORA2A genotypes and sleep variables, as well as the lack of stronger correlations between caffeine load and other sleep variables [16].

In our study, we can suggest that once the C/C genotype carriers of rs5751876 and rs3761422 have taken caffeine, their sleep time will match that of T/T and C/T. Also, when G allele carriers for rs4822492 have taken caffeine, their TST will match the one of C/C, which pertains to the HT4 haplotype of Bodenmann et al. (2012) [14]. The rs5751876 SNP is a synonymous variant (it does not cause an amino acid change in the encoded protein) located on the exon 4 position, while rs2298383 and rs3761422 are located in the 5’UTR, and rs4822492 in 3’UTR, respectively.

We evidenced that both rs2298383, rs3761422, and rs4822492 are in strong linkage disequilibrium with the most studied rs5751876 SNP. Thus, these SNPs may represent potential functional variants [30,31] implicated in the relationship between sleep duration and caffeine consumption. Alternative splicing of the 5’UTR could play a crucial role in the post-transcriptional regulation of G protein-coupled receptors, including the modulation of translational efficiency, message stability, and subcellular localization, and differential expression of 5’UTR splice variants of the adenosine A_2A_ receptor gene was shown in vitro in human stimulated granulocytes in sepsis patients compared to healthy volunteers [32]. The 3’UTR region contains binding sites for regulatory proteins and miRNA which can down-regulate post-transcriptional genes expression by inhibiting protein translation or by destabilizing target transcripts. A second study of Kreth et al. [33] team has described the regulation of A2AR mRNA translation in human polymorphonuclear leukocytes by three miRNA. Barett et al. (2012) [31] reviewed the different mechanism by which non-coding regions, including the 5’ and 3’ UTR, introns, and intergenic regions, are vital for the precise regulation of gene expression.

In our study, TST was significantly lower in participants with the haplotypes 2 and 3 compared with the more frequent protective haplotype 1 (ATCCCG, 45.3% of participants). Haplotype 3, which corresponds to the HT4 haplotype of Bodenmann et al. (2012) [14], confirmed the SNPs analysis for rs5751876 and rs3761422 (T variant sleeping less) and for rs4822492 and rs2298383 (C variant sleeping less). The alleles for rs5751876, rs3761422, rs2298383, and rs4822492 in haplotype 1 are in higher frequency compared to the ones in haplotype 2 and 3.

In this study, we evidenced significant differences between total sleep time (TST) among variants of four SNPs of ADORA2A and two haplotypes but in low caffeine consumers only, and three of them have been previously demonstrated as risk variants for anxious personality (i.e., rs5751876, rs2298383, and rs3761422, in high LD) [17]. In this study, after Bonferroni adjustment for age, sex, and caffeine consumption, the risk haplotype for the anxiety-related personality score (HA1 subdimension, anticipatory worry, and pessimism vs. uninhibited optimism) included alleles rs5751876-T, rs3761422-T, and rs2298383-C. We found two similar haplotypes (2 and 3) associated with lower TST in comparison with the more frequent protective haplotype 1 which suggested that lower TST in low caffeine consumers may be related to anxiety-related personalities.

Moreover, in our study, the risk of sleep complaints was significantly higher for participants carrying the T/T and G/G compared with C/C ancestral genotype for rs2298383 and rs4822492, respectively, and this was observed when considering all subjects and also in moderate caffeine consumers. A recent cross-sectional population-based study on the relationship between depression and symptoms and the rs2298383 SNP for ADORA2A (1253 participants aged 18–35 years, including 228 in current episode of depression) demonstrated that the TT genotype of rs2298383 (compared to CC plus CT) is associated with reduced risk depression and reduced sleep disturbances and less difficulty in concentrating after adjustment for confounding variables such as smoking, gender, socioeconomic class, and ethnicity [21]. This result differs from ours likely because we estimated sleep complaints risk by comparison with the rs2298383 ancestral C/C genotype, as previously described by Hohoff et al. (2010) [17]. Furthermore, our results are consistent regarding genotype frequencies for the six SNPs with those of the 1000 Genomes Project [24] and are consistent with the results on the risk of insomnia.

Indeed, the risk of insomnia is also significantly higher for the T/T compared to the ancestral C/C genotype for rs2298383 and also for G/G carriers of rs4822492, when considering all subjects. In our study, it was difficult to analyze the impact of ADORA2A SNPs on insomnia on the three groups of caffeine consumers because only 10.7% of subjects were identified with insomnia. To our knowledge, the rs4822498 ADORA2A has been identified only once in a genome-wide study on the association between caffeine-induced sleep disturbance and several genetic variants, and the result was inconclusive [19].

With respect to sleep complaints, our results showed no significant difference in sleep complaints prevalence between genotypes in the six ADORA2A SNPs among high caffeine consumers, who are more than half of those affected (52.5%). This result suggests that other genetic mutations may be implicated in the high caffeine dose effect on sleep complaints [34], such as SNPs for ADORA1A. However, multiple variants of the ADORA1A were shown not to be implicated in caffeine-related sleep disturbances [35] nor caffeine-related anxiety (Rogers et al., 2010), and not associated with the in vivo variation of A1AR availability in the human brain compared while ADORA2A rs5751876 was [18]. For us, genetic variants for the cytochrome P450 1A_2_ (CYP1A2), the main system responsible for caffeine metabolism, and variants for pro-inflammatory cytokines should be investigated. Indeed, mutations on the CYP1A2 gene were recently shown to influence abstract reasoning in a large population (1374 participants) under free caffeine intake in everyday life [36], and genetic variations of inflammatory cytokines IL-6 and TNF-α have been associated in several studies with sleep disturbances [37] and susceptibility and severity of obstructive sleep apnea [38,39].

Moreover, several studies have also shown that T/T genotype carriers for rs5751876 of ADORA2A are associated with higher anxiogenic responses to caffeine [12,20]. The differential response of T/T vs. C/C and C/T genotypes was apparent at 100 mg dose of caffeine, and similar results were found for the nearly completely linked SNP rs3761422 and rs2298383 (but not significant after adjustment for multiple testing for the latter) [12]. For Childs et al. (2008) [20], individuals with the C/C genotype for rs2298383 and rs4822492 reported significantly higher anxiety than those with the T/T genotype and G/G, respectively. We can suggest that as soon as caffeine is consumed, anxiety may be implicated in the relationship with sleep duration, particularly in participants carrying the T allele (homozygote and heterozygote) for rs5751876, rs3761422, and rs2298383, and being in the lower frequency C/C genotype for rs4822492.

However, our study also has several limitations that need to be addressed in future studies. We have relied on self-report data for all of the variables analyzed, which can be influenced by perception bias [40]. To this end, it will be important to investigate the impact of ADORA2A SNPs on sleep duration using polysomnography or actigraphy to get an objective measure of sleep. These methods provide greater accuracy of measurement, but they are much more time-consuming and less conducive to our large sample sizes study. However, we confirmed the association between sleep and rs5751876 that was initially discovered in a sample that used polysomnography [15], which suggests that self-report data, while imperfect, is a somewhat reliable measure of the true phenotype and may be of particular use in large-scale genetic studies.

## 4. Materials and Methods

### 4.1. Study Design and Participants

This is a cross-sectional population-based study of active French workers (men and women) aged 18 to 60. The sample consisted of 1023 participants recruited from Hotel Dieu sleep center (APHP), the medical department of EY (First Tower, La defense) and the armed forces biomedical research institute (IRBA). The study received the agreement of the Cochin – CPP Ile de France 1 (Paris) Ethics Committee and was approved by the Agence Nationale pour la Sécurité du Médicament (ANSM) (2017-A00234_49). It was conducted according to the principles expressed in the Declaration of Helsinki of 1975, as revised in 2001 after obtaining written informed consent for all the participants. The study has been registered on the ClinicalTrials.gov public database (NCT number: NCT03855774).

Exclusion criteria were unemployed, retired, medical leave, and pregnant women volunteers, lack of social insurance, and under-18 years old subjects.

### 4.2. Questionnaire

The participants answered a questionnaire for sociodemographic characteristics, assessment of sleep complaints and disorders, and caffeine intake. All data were obtained by means of a paper questionnaire and done at the same time as the saliva sampling. The questionnaire was anonymous, and participants have to refer to the last month to evaluate sleep and wake disorders. The questionnaires had different types of questioning: open-ended, closed-ended, and multiple-choice questions.

#### 4.2.1. Sleep Habits and Complaints

For sleep evaluation, the questionnaire was constructed based on the Sleep Complaints Questionnaire–French version (SDQFV) [41], and the Epworth Sleepiness Scale (ESS) [42].

In addition, sleeping pills, stimulated substances, and other drug consumption were informed in the questionnaire. The SDQFV is a 42-item questionnaire based on the Stanford Sleep Questionnaire and Evaluation of Wakefulness (SQAW). The French version has been validated in several epidemiological studies [41]. It covers sleep habits and complaints. We focused on four specific sleep complaints which are commonly associated with insomnia: (1) difficulties in falling asleep, (2) frequent nocturnal awakenings, (3) early awakenings, and (4) nonrestorative sleep.

The ESS, a self-administered questionnaire, is a subjective tool to assess sleepiness [42]. To assess sleepiness, we also introduced several commonly associated items: (1) napping habits; (2) taking more than five cups of coffee every day; (3) snoring loudly; (4) feeling sleep deprived.

#### 4.2.2. Sleep Complaints

Their definition was based on the International Classification of Sleep complaints (ICSD, 3rd edition) and the Diagnostic and Statistical Manual of Mental Disorders, 4th revision (DSM-IV) [43].

Insomnia was defined by the presence of at least one of the four sleep complaints over at least one month and with daytime consequences. Hypersomnia was measured on a daily basis according to whether patients “fall asleep during the day, during work, while listening to the radio or music, while traveling, in front of the TV” and scored >10 in the ESS. Severe insomnia, regular use of sedatives, and sleep apnea were also considered.

#### 4.2.3. Caffeine Consumption

The questionnaire included the following beverages and caffeine-containing foods: coffee with caffeine, tea, cola, and other carbonated beverages with caffeine, and chocolate. For each item, participants were asked how often, on average, they had consumed a specified amount of each beverage or food over the past year. The participants could choose from nine frequency categories (never, 1–3 per month, 1 per week, 2–4 per week, 5–6 per week, 1 per day, 2–3 per day, 4–5 per day, and 6 or more per day). Typical milligram doses (Mayo Clinic–http://www.mayoclinic.com/health/caffeine/AN01211) were assigned to each and an approximate daily intake was obtained [44]. Based on previous criteria [12,22], participants were then characterized as having low (0–50 mg·day^−1^), moderate (51–300 mg·day^−1^), and high (>300 mg·day^−1^) caffeine intake.

### 4.3. Saliva DNA Extract and Genotyping

Genotypes were determined by investigators who were blind for trait anxiety, sleep and waking EEG, subjective sleepiness, and behavioral results. Oragene DNA kits OG-500 (DNAgenotek, Ottawa, Canada) were used to collect whole saliva samples from healthy adult volunteers (*n* = 1069) after rinsing the mouth with water and at least 30 min after eating or drinking. DNA from saliva collected in Oragene containers are stable for at least five years at ambient temperature. After manual cell lysate preparation, the genomic DNA purification was performed by the Autopure LS instrument (Qiagen, Hilden, Germany). DNA quantity, integrity, and ability to PCR were evaluated by quality controls. One thousand and twenty-three DNA extracts were transferred on 384-well plates. SNP selection was based on previous studies. Participants were genotyped by predesigned or customized probes; TaqMan SNP genotyping assays were provided by Thermo Fisher Scientific (Whaltham, USA). PCR was performed on GeneAmp PCR System 9700, and a 7900HT system with SDS software version 2.4 (Applied Biosystems, Foster City, USA) was used for fluorescence detection and allelic discrimination. A check by Sanger sequencing of PCR products was carried out for rs5751876 because the ADORA2A gene includes many SNPs in this region. The genomic organization of the ADORA2A gene with SNP positions investigated in the 1023 participants is illustrated in Figure 1A.

### 4.4. Statistical Analysis

Statistical tests were performed using R studio (R software (version 3.6.0, 04.24.2019) (www.r-project.org), and significance (α risk) was fixed at *p* < 0.05. Qualitative variables are presented as occurrence and percentage [*n* (%)] and quantitative variables as mean ± 95% CI (confidence interval). The genotype distributions in both alleles were examined using the Hardy–Weinberg equilibrium. The difference in TST was calculated using a 2-way analysis of variance (SNP alleles and caffeine consumption). If a significant interaction was observed, a post-hoc Bonferroni test was used to compare the allele (heterozygote and homozygote mutation) versus the ancestral allele and caffeine consumption (moderate and high versus low) for the 6 SNPs. Qualitative variables (sleep complaints) association with SNP alleles were made using a X^2^ test. Odds ratio adjusted for age, sex, and tobacco use and the 95% confidence intervals (95% CI) were calculated. The SNPStats program [45] was adopted for linkage disequilibrium (D, D, and R square) haplotype analysis. All haplotypes identified were computed, but in order to minimize loss of power, the six haplotypes with a frequency higher than 1% were taken into account for statistical analysis. The most common haplotype was selected as the reference for posthoc analysis.

## 5. Conclusions

In conclusion, our results showed ADORA2A genetic variations related to the duration of nocturnal sleep in a large population of European ancestry aged 18 to 60 years but only in low caffeine consumers (below 50 mg per day, i.e., less than one expresso). As soon as participants became moderate caffeine consumers (51–300 mg per day), the nocturnal sleep decreases whatever the SNPs ADORA2A variants. At caffeine consumption above 300 mg per day, the six genetic variants of ADORA2A studied here do not influence the total sleep time which remains significantly lower compared to low caffeine consumption. These results suggested that caffeine consumption above 300 mg per day may have completely blocked A2A receptors. The herein presented results confirmed previous results on the synonymous rs5751876 variant, and added information on several potential functional variants such as rs3761422, rs2298383 and rs4822492. On the other hand, ADORA2A genetic variations influenced the risk of sleep complaints and insomnia in all caffeine consumers, notably the rs2298383, rs4822492 and rs5751876. Indeed, the risk of sleep complaints is higher in rs2298383 TT and rs4822492 GG compared to the ancestral CC genotypes, and lower in rs5751876 CT and TT (Table 4), and this remained significant in the moderate caffeine consumers. We planned in our future studies to analyze A2A receptors density, function or gene expression in human blood in relation with the three genotypes of rs2298383, rs4822492 and rs5751876 ADORA2A variants. It will be interesting in future studies to investigate in a large population of European ancestry sleep duration and risk of sleep complaints and disorders concomitantly with multiple candidate SNP including dopamine- and cytokines-related genes [39,46], and genes involved in the pharmacokinetic and pharmacodynamic of caffeine effects [35,36]. Also, analysis of changes in A2A receptors density, function or gene expression in human granulocytes under low and high doses of caffeine consumption will aid in understanding the relationship between genetic mutations and sleep complaints and disorders, and will have implications for the treatment of sleep complaints [47].

## Figures and Tables

**Table 1:** The sociodemographic data, lifestyle habits, sleep duration, and sleep disorders of subjects.

Women or Men	405 (39.6%) or 618 (60.4%)
Age, years	32.5 ± 9.6
BMI, kg/m^2^	23.6 ± 3.5
Alcool consumption (min 1 glass/day)	76 (7.4%)
Current smoker (>1 cigarette per day)	192 (18.8%)
Physical activity (>2 h/week)	671 (65.6%)
Shift work (3 × 8 h)	19 (1.9%)
Medication (>5 years)	129 (12.6%)
Caffeine consumption, mg/day	243 ± 208
Epworth sleepiness scale, score	7.7 ± 3.9
Total sleep time, h	7.0 ± 1.0
Sleep complaints (>3 months)	479 (46.8%)
Sleep disorders	
Insomnia	109 (10.7%)
Severe insomnia	4 (0.4%)
Sleep apnea	115 (11.2%)
Use of sedative	9 (0.9%)

Values are mean ± SD (quantitative variable) or occurrence (%) (qualitative variable), *N* = 1023.

**Table 2:** Total sleep time (TST), sleep complaints, and insomnia, according to caffeine consumption.

	Low Caffeine Consumers (0–50 mg/day)	Moderate Caffeine Consumers (51–300 mg/day)	High Caffeine Consumers (>300 mg/day)	Statistical Analysis
*N*	202 (19.7%)	478 (46.7%)	343 (33.5%)	
TST(total sleep time), hours ± SD	7.15 ± 1.16	7.04 ± 0.89	6.75± 0.93 *#	*F* = 13.9, *p* = 0.01
Sleep complaints >3 months, *n* (%)	93 (46.0%)	206 (43.1%)	180 (52.5%) *#	*X*^2^ = 7.1, *p* = 0.03
Insomnia, *n* (%)	15 (7.4%)	51 (10.7%)	43 (12.5%) *	*X*^2^ = 3.5, *p* = 0.05

*N*: number of subjects in the three groups with caffeine consumption. SD: standard deviation; * *p* < 0.01, vs. low caffeine consumers (0–50 mg/day); ^#^ *p* < 0.01 vs. moderate caffeine consumers (51–300 mg/day).

**Table 3:** The six ADORA2A SNPs characteristics and genotypes frequencies.

				Genotype Frequency	
SNP	Gene Position (GRCh38)	Chromosome/Location	Genotype	This Study	1000 Genomes
rs5751862	5’ flank, regulation	22/ 24,406,596	G/G (a) G/A A/A	28.8 48.2 21.9	25 50.5 24
rs2298383	5’UTR	22/ 24,429,543	C/C (a) C/T T/T	20.6 45.3 32.4	17.1 48.5 34.4
rs3761422	5’UTR	22/ 24,430,704	C/C (a) C/T T/T	38.9 44.8 14.6	38.4 46.3 15.3
rs2236624	Intron 3–4	22/ 24,440,056	C/C (a) C/T T/T	61.1 32.8 5.1	54.7 36.8 8.5
rs5751876	Exon 4	22/ 24,441,333	C/C (a) C/T T/T	35 44.5 18.1	37.4 47.1 15.5
rs4822492	3’ UTR (antisense)	22/ 24,447,626	C/C (a) C/G G/G	20.5 45.1 33	17.1 48.5 34.4

(a) ancestral genotype; *N* = 1023 in this study.

**Table 4:** Impact of the six ADORA2A SNPs on total sleep time (TST), sleep complaints, and insomnia.

SNP	Genotype	*N*	Total Sleep Time (TST)	Sleep Complaints		Insomnia	
			Mean ± 95% CI	*n* (%)	ORa (95% CI)	*n* (%)	ORa (95% CI)
**rs5751862**	**G/G (a)**	293	7.02 ± 0.12	124 (42.3%)	1	32 (10.9%)	1
	G/A	493	6.98 ± 0.14	226 (46.0%)	1.2 (0.9–1.5)	46 (9.3%)	1.2 (0.73–2.13)
	A/A	224	6.89 ± 0.16	123 (54.7%)	1.6 (0.9–2.3)	30 (13.4%)	0.8 (0.52–1.34)
**rs2298383**	**C/C (a)**	210	6.99 ± 0.14	88 (41.9%)	1	23 (10.9%)	1
	C/T	463	6.94 ± 0.12	207 (44.7%)	1.2 (0.6–1.4)	40 (8.6%)	0.8 (0.4–1.3)
	T/T	332	7.01 ± 0.14	174 (52.4%)	1.9 (1.2–3.1) *	44 (13.3%)	1.5 (1.3–2.5) *
**rs3761422**	**C/C (a)**	398	6.99 ± 0.10	200 (49.1%)	1	49 (12.0%)	1
	C/T	458	6.91 ± 0.10	204 (44.5%)	0.7 (0.6–1.0)	38 (8.3%)	0.6 (0.4–0.9) *
	T/T	149	7.08 ± 0.16	64 (43.0%)	0.8 (0.5–1.1)	19 (12.8%)	0.6 (0.6–1.7)
**rs2236624**	**C/C (a)**	625	6.97 ± 0.04	299 (47.8%)	1	67 (10.7%)	1
	C/T	337	6.94 ± 0.05	153 (45.4%)	0.9 (0.7–1.2)	35 (10.4%)	0.9 (0.6–1.6)
	T/T	51	7.03 ± 0.14	21 (41.2%)	0.8 (0.4–1.4)	7 (13.7%)	1.4 (0.6–3.2)
**rs5751876**	**C/C (a)**	359	6.98 ± 0.05	185 (51.5%)	1	46 (12.8%)	1
	C/T	454	6.93 ± 0.04	198 (43.6%)	0.7 (0.5–0.9) *	36 (7.9%)	0.5 (0.3–0.9) *
	T/T	182	6.99 ± 0.08	80 (43.2%)	0.7 (0.5–0.9) *	21 (11.5%)	0.9 (0.5–1.5)
**rs4822492**	**C/C (a)**	210	6.98 ± 0.05	89 (42.4%)	1	23 (10.9%)	1
	C/G	461	6.93 ± 0.04	207 (44.9%)	1.1 (0.5–1.2)	39 (8.4%)	1.3 (0.7–2.2)
	G/G	339	7.01 ± 0.07	178 (52.5%)	1.5 (1.1–2.1) *	46 (13.6%)	1.9 (1.3–3.2) *

A = ancestral. * *p* < 0.05 significant difference vs. the ancestral genotype (C/C for rs5751876, rs2298383, rs3761422, and rs4822492). *N* is the total number of subjects with the alleles, *n* is the number of subjects with sleep complaints and insomnia for each genotype. ORa: adjusted odds ratio (age, tobacco, sex).

**Table 5:** Impact of the six ADORA2A SNPs on total sleep time (TST) according to caffeine consumption.

SNP	Genotype	Low Caffeine Consumers (0–50 mg/day)		Moderate Caffeine Consumers (51–300 mg/day)		High Caffeine Consumers (>300 mg/day)		*ANOVA (F (p))*		
		*N*	Mean ± 95% CI	*N*	Mean ± 95% CI	*N*	Mean ± 95% CI	Caffeine Groups (F_2,1021_)	Genotype (F_2,1021_)	Interaction (F_4,1019_)
rs5751862	G/G (a)	52 (17.8%)	7.23 ± 0.44	148 (50.5%)	7.09 ± 0.22	93 (31.7%)	6.95 ± 0.14	7.4 (*p* =< 0.001)	0.2 (*p* = 0.78)	1.2 (*p* = 0.26)
	G/A	106 (21.5%)	7.28 ± 0.26	223 (45.1%)	7.10 ± 0.14 #	164 (33.1%)	6.81 ± 0.12 #			
	A/A	39 (17.3%)	7.46 ± 0.44	102 (45.3%)	6.94 ± 0.18	83 (36.9%)	6.77 ± 0.18 #			
rs2298383	C/C (a)	62 (18.7%)	6.93 ± 0.44	155 (46.8%)	7.10 ± 0.14	114 (34.4%)	6.96 ± 0.18	8.2 (*p* < 0.001)	0.2 (*p* = 0.81)	1.9 (*p* = 0.03)
	C/T	92 (19.8%)	7.13 ± 0.44	198 (42.7%)	7.09 ± 0.14	172 (37.1%)	6.79 ± 0.12 #			
	T/T	44 (20.9 %)	7.52 ± 0.32 *	113 (53.8%)	6.99 ± 0.16 #	53 (25%)	6.88 ± 0.14 #			
rs3761422	C/C (a)	79 (19.3%)	7.56 ± 0.28	187 (45.8%)	7.00 ± 0.16 #	131 (32.1%)	6.84 ± 0.14 #	7.1 (*p* =< 0.001)	0.4 (*p* = 0.66)	1.2 (*p* = 0.04)
	C/T	89 (19.4%)	7.00 ± 0.32 *	204 (44.5%)	7.06 ± 0.14	164 (35.8%)	6.79 ± 0.12 #			
	T/T	28 (18.8%)	6.85 ± 0.45 *	76 (51.0%)	7.27 ± 0.32	45 (30.2%)	6.99 ± 0.10 #			
rs2236624	C/C (a)	130 (20.8%)	7.30 ± 0.26	284 (45.4%)	7.00 ± 0.12 #	210 (33.6%)	6.86 ± 0.10 #	3.5 (*p* = 0.002)	0.3 (*p* = 0.73)	1.4 (*p* = 0.18)
	C/T	60 (19.6%)	7.17 ± 0.36	159 (47.2%)	7.21± 0.20	117 (34.7%)	6.74 ± 0.14 #			
	T/T	11(21.6%)	6.62 ± 0.80	27 (52.9%)	7.12 ± 0.32	13 (25.4%)	7.05 ± 0.28 #			
rs5751876	C/C (a)	68 (18.9%)	7.53 ± 0.30	165 (46.0%)	6.99 ± 0.14 #	125 (34.8%)	6.85 ± 0.14 #	7.5 (*p* < 0.001)	0.1 (*p* = 0.91)	1.8 (*p* = 0.04)
	C/T	91 (20.0%)	7.05 ± 0.32 *	199 (36.5%)	7.09 ± 0.20	163 (35.9%)	6.80 ± 0.12 #			
	T/T	38 (20.9%)	6.92 ± 0.48 *	98 (53.8%)	7.01 ± 0.28	49 (26.9%)	6.97 ± 0.20			
rs4822492	C/C (a)	44 (21.0%)	6.93 ± 0.44	113 (53.8%)	6.99 ± 0.14	53 (25.2%)	6.86 ± 0.14	8.2 (*p* < 0.001)	0.1 (*p* = 0.89)	1.8 (*p* = 0.04)
	C/G	91 (19.7%)	7.26 ± 0.32 *	200 (43.3%)	7.09 ± 0.14	169 (36.7%)	6.79 ± 0.12 #			
	G/G	64 (18.9%)	7.52 ± 0.30*	159 (46.9%)	7.10 ± 0.24 #	115 (33.9%)	6.96 ± 0.18 #			

A = ancestral. * *p* < 0.05 significant difference vs. the ancestral genotype (C/C for rs5751876, rs2298383, rs3761422, and rs4822492). # *p* < 0.05 versus low caffeine consumers (0–50 mg/day). *N* is the total number of subjects with the alleles, *n* is the number of subjects with sleep complaints and insomnia for each genotype (% are calculated according to the number of subjects in each caffeine group; see Table 5). F_2,1021_ and F_2,1019_ are results of the 2-way ANOVA (caffeine consumption groups X SNP alleles) analysis.

**Table 6:** The ADORA2A haplotypes frequencies (*n* = 1023 participants).

	rs5751862	rs2298383	rs3761422	rs2236624	rs5751876	rs4822492	%
1	A	T	C	C	C	G	45.3%
2	G	C	T	T	T	C	21.2%
3 ^a^	G	C	T	C	T	C	15.7%
4	G	T	C	C	C	G	11.1%
5	G	C	C	C	T	C	2.8%
6	G	C	C	C	C	C	2.6%
Rare							1.30%

Rare are haplotypes <1%; ^a^ corresponding to HT4 of Bodenmann et al. (2012) [14].

**Table 7:** Impact of ADORA2A haplotypes on total sleep time (TST) according to caffeine consumption.

TST (h)					
Haplotype	All Subjects	Low Caffeine Consumers (0–50 mg/day)	Moderate Caffeine Consumers (51–300 mg/day)	High Caffeine Consumers (>300 mg/day)	Caffeine Consumption Effect
ATCCCG	6.81 ± 0.08	7.54 ± 0.1	6.99 ± 0.14#	6.60 ± 0.18 #	*p* < 0.001
GCTTTC	6.63 + 0.19	6.92 ± 0.25*	6.88 ± 0.17	6.46 ± 0.28#	*p* < 0.01
GCTCTC	6.70 + 0.17	7.04 ± 0.26*	6.89 ± 0.34	6.55 ± 0.23#	*p* < 0.05
GTCCCG	6.87 + 0.18	7.38 ± 0.32	7.05 ± 0.38#	6.69 ± 0.3#	*p* < 0.001
GCCCTC	6.83 + 0.4	7.51 ± 0.27	7.05 ± 0.28#	6.60 ± 0.52#	*p* < 0.001
GCCCCC	6.76 + 0.23	7.30 ± 0.27	7.03 ± 0.42#	6.54 ± 0.42#	*p* < 0.001
Rare	6.79 + 0.46	7.31 ± 0.57	7.01 ± 0.62	6.60 ± 0.62#	*p* < 0.001

Mean ± 95% CI; * versus ATCCCG (most frequent haplotype 1); # versus low caffeine consumers (0–50 mg/day).

**Table 8:** Impact of the six ADORA2A SNPs on sleep complaints according to caffeine consumption.

SNP	Genotype	Low Caffeine Consumers (0–50 mg/day)		Moderate Caffeine Consumers (51–300 mg/day)		High Caffeine Consumers (>300 mg/day)	
		*n* (%)	ORa (95% CI)	*n* (%)	ORa (95% CI)	*n* (%)	ORa (95% CI)
**rs5751862**	G/G (a)	21 (40.4%)	1	57 (37.3%)	1	56 (52.3%)	1
	G/A	52 (49.1%)	1.5 (0.6–3.9)	96 (42.1%)	1.3 (0.9–2.6)	79 (49.4.5%)	0.85 (0.5–1.2)
	A/A	18 (46.2%)	1.3 (0.9–8.7)	59 (55.7%)	1.4 (0.8–2.1)	46 (57.5%)	1.27 (0.8–2.0)
**rs2298383**	C/C (a)	19 (43.2%)	1	44 (38.9%)	1	24 (45.3%)	1
	C/T	46 (50.6%)	1.3 (0.6–2.7)	71 (35.9%)	1.1 (0.7–1.8)	87 (50.6%)	1.1 (0.7–1.8)
	T/T	26 (41.9%)	0.9 (0.6–1.6)	85 (54.8%)	1.5 (1.1–2.8) *	60 (52.6%)	1.3 (0.9–2.9)
**rs3761422**	C/C (a)	35 (44.3%)	1	100 (51.8%)	1	65 (51.6%)	1
	C/T	44 (49.4%)	1.3 (0.3–1.6)	74 (35.4%)	0.7 (0.4–1.1)	86 (53.8.%)	1.1 (0.6–1.6)
	T/T	11 (39.3%)	0.3 (0.1–1.1)	33 (41.8%)	0.7 (0.4–1.4)	20 (47.6%)	0.9 (0.5–1.5)
**rs2236624**	C/C (a)	59 (45.4%)	1	132 (45.5%)	1	108 (52.7%)	1
	C/T	31 (51.7%)	1.1 (0.5–2.6)	63 (38.4%)	0.9 (0.6–1.4)	59 (52.2%)	0.9 (0.6–1.2)
	T/T	2 (18.2%)	0	14 (48.3%)	0.9 (0.3–2.2)	5 (45.5%)	1.1 (0.5–2.5)
**rs5751876**	C/C (a)	31 (45.6%)	1	90 (52.6%)	1	64 (56.3%)	1
	C/T	43 (47.3%)	1.2 (0.6–2.6)	72 (35.3%)	0.6 (0.4–0.9) *	83 (52.2%)	0.9 (0.6–1.2)
	T/T	17 (44.7%)	0.8 (0.3–1.6)	41 (49.6%)	0.8 (0.3–1.2)	22 (47.8%)	0.8 (0.5–1.3)
**rs4822492**	C/C (a)	19 (43.2%)	1	45 (39.8%)	1	24 (45.3.%)	1
	C/G	46 (50.5%)	0.7 (0.3–1.6)	72 (36.0%)	0.8 (0.5–1.6)	86 (50.9%)	1.1 (0.6–2.3)
	G/G	28 (43.8%)	1.0 (0.4–2.1)	87 (54.7%)	1.8 (1.1–2.9) *	61 (53.0%)	1.3 (0.6–2.5)

(a) = ancestral. * *p* < 0.05 significant difference vs. the ancestral genotype; CI; * versus ATCCCG (most frequent haplotype 1).
